# Machine learning predicts the risk of osteoporosis in patients with breast cancer and healthy women

**DOI:** 10.1007/s00432-024-05622-8

**Published:** 2024-02-23

**Authors:** Fang Zhao, Chaofan Li, Weiwei Wang, Yu Zhang, Peizhuo Yao, Xinyu Wei, Yiwei Jia, Shaonong Dang, Shuqun Zhang

**Affiliations:** 1 Department of Oncology, The Second Affiliated Hospital of Xi’an Jiaotong University, 157 West Fifth Street, Xi’an, Shaanxi People’s Republic of China; 2 Department of Epidemiology and Biostatistics, School of Public Health, Xi’an Jiaotong University Health Science Center, Xi’an, 710061 Shaanxi People’s Republic of China

**Keywords:** Breast cancer, Machine learning, Body composition, Bone density, Bone metabolism indicators

## Abstract

**Objective:**

In this study, we investigated the effects of endocrine therapy and related drugs on the body composition and bone metabolism of patients with breast cancer. Additionally, using body composition-related indicators in machine learning algorithms, the risks of osteoporosis in patients with breast cancer and healthy women were predicted.

**Methods:**

We enrolled postmenopausal patients with breast cancer who were hospitalized in a tertiary hospital and postmenopausal women undergoing health checkups in our hospital between 2019 and 2021. The basic information, body composition, bone density-related indicators, and bone metabolism-related indicators of all the study subjects were recorded. Machine learning models were constructed using cross-validation.

**Results:**

Compared with a healthy population, the body composition of patients with breast cancer was low in bone mass, protein, body fat percentage, muscle, and basal metabolism, whereas total water, intracellular fluid, extracellular fluid, and waist-to-hip ratio were high. In patients with breast cancer, the bone mineral density (BMD), *Z* value, and *T* value were low and the proportion of bone loss and osteoporosis was high. BMD in patients with breast cancer was negatively correlated with age, endocrine therapy status, duration of medication, and duration of menopause, and it was positively correlated with body mass index (BMI) and basal metabolism. The parameters including body composition, age, hormone receptor status, and medication type were used for developing the machine learning model to predict osteoporosis risk in patients with breast cancer and healthy populations. The model showed a high accuracy in predicting osteoporosis, reflecting the predictive value of the model.

**Conclusions:**

Patients with breast cancer may have changed body composition and BMD. Compared with the healthy population, the main indicators of osteoporosis in patients with breast cancer were reduced nonadipose tissue, increased risk of edema, altered fat distribution, and reduced BMD. In addition to age, duration of treatment, and duration of menopause, body composition-related indicators such as BMI and basal metabolism may be considerably associated with BMD of patients with breast cancer, suggesting that BMD status can be monitored in clinical practice by focusing on changes in the aforementioned indexes, which may provide a way to prevent preclinical osteoporosis.

**Supplementary Information:**

The online version contains supplementary material available at 10.1007/s00432-024-05622-8.

## Introduction

Breast cancer has surpassed lung cancer to become the most prevalent cancer worldwide and has the highest morbidity and mortality rates for women (Sung et al. 2021). Some studies have reported that patients with breast cancer are often accompanied by bone metabolism disorders, including decreased bone mineral density (BMD) and imbalance between bone formation and bone resorption in bone metabolism indexes, with bone resorption being the most common. Reduced bone density is mainly due to perforation of the trabecular plate, loss of trabecular connectivity, and reduced trabecular mass, which results in an overall decrease in bone strength, leading to bone loss, osteoporosis, and even osteoporotic fracture. This has been confirmed in several clinical studies, a meta-analysis that included 30 studies revealed that aromatase inhibitor (AI)-treated patients with breast cancer had a 35% increase in osteoporotic fractures, an 18% increase in hip fractures, an 84% increase in vertebral fractures, and an 18% increase in non-vertebral fractures (Lee et al. 2020). Breast cancer-related clinical trials (including NSABP B-42, DATA, IDEAL, MA.17R, and ABCSG-16) that compared the effects of extended duration of AI versus placebo or no treatment further confirmed an increased risk of fracture owing to AI use (Hellemond et al. 2018, 2019; Mamounas et al. 2019). The high cost of treatment and care for osteoporosis and osteoporotic fractures creates a heavy financial burden and induces psychological stress on patients, along with a considerable economic burden on society. With aging, disease progression, and drug interventions, body composition changes accordingly; e.g., body fat percentage, body mass index (BMI), and waist-to-hip ratio can reflect the degree of obesity. Determination of body composition, which is of great importance in evaluating the nutritional status of the human body, the efficacy of disease treatment, obesity management, and health maintenance. Some studies (Napoli et al. 2013; Liu et al. 2022; Hamood et al. 2018) have shown that changes in body composition have a certain correlation with breast cancer treatment and prognosis of the patients, and that weight gain, obesity, and edema are detrimental to the prognosis of patients with breast cancer.

However, most studies have been conducted on European and American patients with breast cancer, and only a few studies have reported the effects of breast cancer treatment on body composition in the Asian population. Previous studies have only focused on body composition or BMD/bone metabolism indexes, and only a few studies combined both to conduct systematic analyses. Studies on the prediction of osteoporosis risk based on body composition data of patients with breast cancer and healthy populations are lacking. Therefore, herein, we systemically analyzed the body composition and bone metabolism of patients with breast cancer in comparison with those of a normal control group (a healthy medical check-up population). Furthermore, the new approach of machine learning has been widely used in various predictions for breast cancer patients (Li et al. 2023 Jun 21; Li et al. 2023 Jun 15; Li et al. 2022 Sep). We explored the effects of endocrine therapy and related drugs on the body composition and bone metabolism of patients with breast cancer and constructed a machine learning model for predicting the risk of osteoporosis in patients with breast cancer and healthy women.

## Methods

### Data sources and study design

The study population included patients with breast cancer hospitalized in the oncology department of a tertiary care hospital between 2019 and 2021, and their data were obtained. Additionally, data on women undergoing health checkups in our hospital during the same period were also collected. A retrospective survey research design was used to investigate the body composition- and bone metabolism-related information during the treatment period of patients undergoing endocrine therapy.

### Inclusion criteria

The inclusion criteria for patients from the tertiary care hospital were as follows: (1) patients who were 40–79 years old, and we included age as a continuous variable in multivariate analyses; (2) patients who were diagnosed with breast cancer via postoperative pathology and confirmed hormone receptor positivity by immunohistochemistry; (3) patients who had completed surgery and/or (neo)adjuvant chemotherapy and/or radiotherapy, along with endocrine therapy (endocrine therapy group), or patients who completed chemotherapy (non-endocrine therapy group) for > 6 months; and (4) patients who were menopausal.

The inclusion criteria for the female patients who underwent health checkups in our hospital were as follows: (1)patients who were aged 40–79 years old, and we included age as a continuous variable in multivariate analyses; (2) patients who were menopausal.

### Exclusion criteria

The exclusion criteria for patients from the tertiary care hospital were as follows: (1) patients who had advanced breast cancer, and advanced breast cancer refers to breast cancer with distant metastases; (2) patients who had other systematic serious diseases, including renal, hepatic, and cardiovascular diseases, and endocrine metabolic diseases, including diabetes mellitus and hyperthyroidism, in combination with breast cancer; (3) patients who had long-term glucocorticoid treatment; (4) patients who were bed-ridden or with severe complications such as edema and ascites that affected measurement; (5) patients who had other diseases affecting bone metabolism; and (6) patients with a history of spinal or extremity long-bone fracture in recent years (≤ 5 years).

The exclusion criteria for the female patients who underwent health checkups in our hospital were the same as the aforementioned criteria numbered 2–6.

### Sample size estimation

Previous studies have reported some methods for calculating sample size (Rock et al. 2022; Peng et al. 2023). For example, the BMD of postmenopausal patients with breast cancer is 0.95 ± 0.07 kg, and it decreases by approximately 0.1 kg after endocrine therapy. Based on this, the sample size was calculated using EPITOOLS; at a certainty of 0.95 and a two-sided confidence interval of 95%, the sample size was calculated to require a minimum of 81 samples in each group. Furthermore, considering a 10% loss-to-follow-up rate, a minimum of 90 patients each was required to be enrolled in the endocrine and non-endocrine treatment groups; hence, a minimum of 180 patients were required to be enrolled.

### Study groups and endocrine treatment protocols

The study was divided into the following three parts: first, comparing and analyzing the differences in the body composition and bone metabolism between the non-endocrine therapy group (112 patients) and healthy medically examined female patients (546 patients); second, comparing and analyzing the body composition and bone metabolism between the endocrine therapy group (229 patients) and non-endocrine therapy group (112 patients); and third, comparing and analyzing the differences in the effects of treatment with tamoxifen (TAM) (92 patients) and treatment with AI (137 patients) on body composition and bone metabolism in patients with breast cancer. Patients in the non-endocrine treatment group have not been treated with TAM or AI, but they received completed surgery and chemotherapy/radiotherapy cycles. The endocrine therapy group refers to female breast cancer patients who have received not only completed surgery and chemotherapy/radiotherapy cycles, but also anti-estrogen/progestin therapy. These patients may have been treated with tamoxifen (TAM) or an aromatase inhibitor (AI) analog (according to whether they are menopausal or not).

The patients with breast cancer who had completed surgery and chemotherapy/radiotherapy cycles were regularly followed up in the outpatient clinic with endocrine medications. TAM was administered through regular TAM citrate tablets (Shandong Health Pharmaceutical Co., Ltd.) at a dosage of 10 mg/tablet, one tablet each time, twice a day. AI was administered through regular anastrozole tablets (AstraZeneca [China] Pharmaceutical Co., Ltd.) at a dosage of 1 mg/tablet, one tablet each time, twice a day, or letrozole tablets (Novartis Pharma AG, Switzerland) at a dose of 2.5 mg/tablet, one tablet each time, once a day.

### Body composition-related indicators and their measurement methodology

Body composition refers to the proportion of each substance that makes up the body, including fat, bone mass, protein, total water, intracellular fluid, extracellular fluid, water ratio, muscle, basal metabolism, body weight, body fat percentage, BMI, and waist-to-hip ratio. It is a collection of several variables. Usually, obesity not only affects the treatment effect in patients with breast cancer but also their bone metabolism. Herein, we assessed the degree of obesity through the following three indicators: body fat percentage, BMI, and waist–hip ratio. The body composition measurements of patients with breast cancer who met the inclusion criteria were performed using a body composition analyzer model BCA-2A (Tsinghua Tongfang) at 20–25 °C. The working principle of BCA-2A Body Composition Analyzer is based on Bioelectrical Impedance Analysis (BIA), which is a method of measuring the electrical impedance of various parts of the human body through the connection of electrodes on the hands and feet of the human body, using different frequency bands of weak (imperceptible to the human body) constant alternating currents, by treating the human body as a conductor. Finally, it is a non-invasive method for assessing the composition of the human body by measuring the amount of fat, bone, protein, muscle, and water in the body based on the different electrical conductivity of different tissues and organs of the human body. Before measurements, the patients were ensured to have an empty stomach, defecate, take off their coats, and remove watches, rings, necklaces, and other metal objects. The patients were requested to stand on the body composition analyzer in an upright position, looking straight ahead, with the heels of the feet close together, the toes of the feet at 45°, the arms at approximately 30°, with the hands and feet fully in contact with the electrodes, and avoid shaking during the testing process. The body composition instrument measurement handling and data entry was done by the staff of our hospital after uniform training. After the measurement, the general personal data were entered, and the instrument system collected the data and automatically generated all the aforementioned parameters, of which, the accuracy of the measurements of fat, bone mass, protein, the contents of total water, intracellular fluid, and extracellular fluid, muscle, and body weight was 0.01 kg. Water ratio, body fat percentage, and waist-to-hip ratio were measured with an accuracy of 0.01, basal metabolism was measured with an accuracy of 0.01 J, and BMI was measured with an accuracy of 0.01 kg/m^2^.

**Table:** 

Body composition	Units	Accuracy
Fat	(kg)	0.01 kg
Bone mass	(kg)	0.01 kg
Protein	(kg)	0.01 kg
Total water	(kg)	0.01 kg
Intracellular fluid	(kg)	0.01 kg
Extracellular fluid	(kg)	0.01 kg
Water ratio	(%)	0.01
Muscle	(kg)	0.01 kg
Basal metabolism	(J)	0.01 J
Body weight	(kg)	0.01 kg
Body fat percentage	(%)	0.01
BMI	(kg/m^2^)	0.01 kg/m^2^
Waist-to-hip ratio	(%)	0.01

### Bone density-related indicators and their measurement methodology

Bone density-related indicators include BMD, *T* value, and *Z* value. BMD refers to the amount of mineral per unit area of a bone, which is an important indicator of the bone mass. BMD T value measurement is the gold standard diagnostic strategy for osteoporosis, as recommended by the World Health Organization. The BMD values of the cancellous bone of lumbar vertebrae 2–4 were measured using the PRIMUS dual-energy X-ray bone densitometer (OsteoSys, South Korea), and the average values were calculated. The bone density was measured as follows: Process calibration was prescanned. The personal information was registered. The examinee was laid flat in the center of the bed for hip positioning with the toes of both feet internally rotated, and the infrared laser measurement point was adjusted to the pubic symphysis joint parallel to the thigh bone at the lower three fingers. Scanning was performed. The analytical interface started after the scanning was complete, and the report was saved and printed. The basic principle of a dual-energy X-ray bone densitometer is to use the dual-energy X-ray absorption method, which includes the use of X-rays, emitted from the X-ray tube, after absorption and filtration, the continuous energy of X-rays into two kinds of high and low energy of X-rays, the rays through the human body, respectively. The intensities of the rays passing through the bones and soft tissues were measured, and based on the absorption characteristics of different energies, the BMD values of the bones and soft tissues were calculated.

Osteoporosis was diagnosed based on the *T* value from the dual-energy X-ray absorptiometry measurement, which is the standard deviation above ( +) or below ( −) the BMD value of a same-sex young population relative to the BMD value of a same-sex normal young population. Patients with a T value of ≤  − 2.5 were diagnosed with osteoporosis. The *Z* value, on the other hand, compares the detected BMD values with those of people of the same sex and age. Although the Z value has a limited role in the diagnosis of osteoporosis, it can reflect the severity of osteoporosis.

### Statistical analysis

Data were analyzed and statistically processed using SPSS 26.0 statistical software. Data that complied with normal distribution were used for measurement data, and a *t*-test or ANOVA was used for comparing groups. Count data are expressed as constitutive ratios. Statistical significance was confirmed using the *χ*2 test, and a *P*-value of < 0.05 was considered statistically significant. Sensitivity analyses were performed using linear regression models to adjust for the effects of covariates and verify the stability of the results.

Multiple stepwise linear regression analyses were performed to investigate the effects of study variables on lumbar bone mineral density (BMD) in patients with breast cancer during endocrine therapy. The significant levels of the entry and exclusion methods of the independent variables were set at 0.05 and 0.10, respectively.

### Machine learning model

For feature selection, patients were randomly divided into training and test groups at a ratio of 7:3. Various features were added to our machine learning model for predicting the risk of osteoporosis in patients with breast cancer and healthy women, including age, breast cancer, estrogen receptor (ER) status, endocrine therapy drugs, radiotherapy, fat mass, bone mass, protein, total water, intracellular fluid, extracellular fluid, water ratio, muscle mass, body weight, body fat percentage, body mass index, waist-to-hip ratio, and basal metabolic rate. Before initiating the training procedure, we obtained response variables for osteoporosis data, wherein 1 indicated osteoporosis and 0 indicated no osteoporosis. Parameter tuning was performed by a random search. In the test data, we compared the area under the curve (AUC) values predicted by logistic regression (LR), random forest (RF), decision tree (ID3), and artificial neural network (ANN). Logistic regression is a classification algorithm, which is also known as log dominance regression (Domínguez-Almendros et al. 2011). Logistic regression is a highly interpretable algorithm and a hallmark of classical predictive modeling. ID3 uses nodes to represent input factors and leaves to represent decision outcomes (Kourou et al. 2015). Through ID3, k samples were repeatedly and randomly drawn from the original training sample set *N*, and then *k* classification trees were generated based on the self-help sample set to generate a random forest. An ANN simulates the behavioral characteristics of animal neural networks and mathematical models of algorithms for distributed parallel information processing (Anderson 1988). This network depends on the complexity of the system to process information by adjusting the relationship between several nodes interconnected within it and can learn and adapt itself. We have provided the R code in the supplemental file to ensure reproducibility.

## Result

### Body composition and bone density status of patients with breast cancer

#### Basic conditions of the study population

We included 112 patients with breast cancer and 546 healthy individuals to evaluate the body composition and bone density characteristics of patients with breast cancer. The average age of the individuals in the two groups was 53.76 and 54.6, the average weight was 60.8 kg and 60.1 kg, and the average height was 159.0 cm and 160.0 cm, respectively. No statistically significant difference was found between the two groups in terms of age, weight, and height. (Table 1).

**Table 1:** Basic information on healthy individuals and patients with breast cancer ($\overline{x}\pm \mathrm{s }$)

	Healthy individuals (*n* = 546)	Patients with breast cancer (*n* = 112)	*t*	*P*
Age (years)	53.12 ± 8.83	53.73 ± 9.99	0.97	0.329
Weight (kg)	60.10 ± 8.52	60.75 ± 8.02	0.74	0.460
Height (cm)	160.0 ± 6.5	159.0 ± 14.0	0.99	0.286

### Body composition analysis of patients with breast cancer and healthy individuals

Compared with healthy individuals, patients with breast cancer exhibited low protein (*P* < 0.001), muscle content (*P* < 0.001), basal metabolism rate (P < 0.001), and high total water (P = 0.036), intracellular fluid (P < 0.001), extracellular fluid (P < 0.001), and water ratio (P = 0.002); and in obesity-related indexes, patients with breast cancer showed high BMI (P = 0.018) and waist-to-hip ratio (P = 0.031). All of the aforementioned indexes were statistically different; however, bone mass, fat, body weight, and percentage of body fat were not statistically different between the two groups. The results are shown in Table 2.

**Table 2:** Comparison of body composition in healthy individuals and patients with breast cancer ($\overline{{\text{x}}}\pm \mathrm{s }$)

Body composition	Healthy individuals (*n* = 546)	Patients with breast cancer (*n* = 112)	*t*	*P*
Lipid (kg)	19.83 ± 5.86	19.89 ± 5.46	− 0.09	0.929
Bone (kg)	2.77 ± 0.29	2.81 ± 0.23	− 1.11	0.199
Protein (kg)	10.85 ± 0.84	8.56 ± 0.89	25.17	< 0.001
Total water (kg)	29.64 ± 3.12	30.32 ± 3.16	− 2.10	0.036
Intracellular fluid (kg)	18.17 ± 1.94	19.09 ± 3.19	− 4.02	< 0.001
Extracellular fluid (kg)	11.47 ± 1.21	13.05 ± 9.23	− 3.86	< 0.001
Water content ratio	0.39 ± 0.06	0.42 ± 0.04	− 3.16	0.002
Muscle (kg)	29.03 ± 2.77	27.80 ± 2.96	5.43	< 0.001
Basic metabolism (BMD) (J)	1239.61 ± 91.50	1186.67 ± 105.49	5.43	< 0.001
Weight (kg)	60.10 ± 8.52	60.75 ± 8.02	− 0.74	0.442
Body fat percentage (%)	32.46 ± 5.90	31.83 ± 5.04	1.06	0.243
BMI (kg/m^2^)	23.55 ± 3.18	24.34 ± 3.23	− 2.38	0.018
Waist–hip ratio	0.90 ± 0.05	0.91 ± 0.06	− 2.16	0.031

### Analysis of bone density-related indexes of patients with breast cancer and healthy individuals

#### Comparison of BMD-related indexes between the two groups of people

Compared with the healthy individuals, the bone density-related indexes of patients with breast cancer exhibited low *T* value (P < 0.001), high BMD value (P = 0.010), and high Z value (P = 0.023), with statistically significant differences. (Table 3).

**Table 3:** Comparison of bone density-related indicators in healthy individuals and patients with breast cancer ($\overline{x}\pm \mathrm{s }$)

	Healthy individuals (*n* = 546)	Patients with breast cancer (*n* = 112)	*t*	*P*
BMD (g/m^2^)	0.98 ± 0.46	1.86 ± 0.83	2.58	0.010
*Z* value	− 0.98 ± 1.34	0.12 ± 1.20	− 8.24	0.023
*T* value	− 0.33 ± 1.14	− 0.97 ± 1.26	5.32	< 0.001

#### Adjustment analysis of bone density-related indexes of the two groups

To eliminate the effect of age and height on the bone density-related indexes of the two groups, the bone density-related indexes of healthy individuals and patients with breast cancer were compared after adjusting the aforementioned factors using multiple regression analysis.

After adjustment, compared with healthy individuals, patients with breast cancer exhibited lower *T* value (*β* =  − 0.55, 95% CI: − 0.85 to − 0.37) (*P* < 0.001) and lower *Z* value (*β* =  − 1.58, 95% CI: − 1.83 to − 1.34) (*P* < 0.001), with statistically significant differences. Furthermore, the BMD was lower, without statistically significant.

BMD, *Z* value, and *T* value, which were statistically different between the two groups before adjusting for age and height factors, were low and statistically significant after adjusting *Z* value and *T* value among patients with breast cancer; however, BMD was low but the difference was not statistically significant. (Table 4).

**Table 4:** Comparison of bone density-related indicators in adjusted healthy individuals and patients with breast cancer

	*β*	95% CI
BMD (g/m^2^)	− 0.08	− 0.02 to 0.17
*Z* value	− 1.58***	− 1.83 to − 1.34
*T* value	− 0.55***	− 0.85 to − 0.37

Note: Linear regression modeling was used to adjust for age and height; healthy individuals were the control; ***P < 0.001

### Body composition, bone density, and bone metabolism in patients with breast cancer receiving endocrine therapy

#### Basic conditions of study participants

In this study, 341 patients with breast cancer were included, of whom 229 received endocrine treatment and 112 did not receive endocrine treatment. The body composition, bone density, and bone metabolism status of patients with breast cancer receiving endocrine treatment were analyzed by comparing the two groups.

The average age of the patients in the two groups was 52.7 and 54.6, respectively. The proportion of the main surgical methods was radical or modified radical surgery, which was 72.5% and 78.6%, respectively, and the proportion of breast-conserving or reconstructive surgery was 15.7% and 14.3%, respectively; whereas the proportion of simple mastectomy was 11.79% and 7.1%, respectively. The pathology of the patients was mainly invasive ductal carcinoma of the common type, which was 87.3% and 83.3%, respectively. The proportion of the patients who received endocrine therapy was 87.3% and 83.3%, respectively. The proportion of the patients who received radiotherapy was 55.5% and 56.3%, respectively. The differences between the two groups in terms of age, choice of surgical method, pathological type, and whether to receive radiotherapy were not statistically significant (Table 5).

**Table 5:** Basic information of patients in the endocrine treatment group and non-endocrine treatment group

Variables	Endocrine (*n* = 229)	Non-endocrine (*n* = 112)	*t*/*χ*^2^	*P*
Age (years) $(\overline{x}\pm \mathrm{s }$)	52.71 ± 10.37	54.60 ± 8.84	0.73	0.057
Surgical Procedures (*n*, %)			2.06	0.357
Radical mastoidectomy or Modified radical mastoidectomy	166 (72.49)	88 (78.57)		
Breast-conserving surgery or breast reconstruction	36 (15.72)	16 (14.29)		
Simple mastectomy	27 (11.79)	8 (7.14)		
Pathological type (*n*, %)			1.83	0.400
Noninvasive breast cancer	17 (7.42)	8 (7.14)		
Nonspecific invasive breast cancer	200 (87.34)	94 (83.93)		
Specific invasive breast cancer	12 (5.24)	10 (8.93)		
Radiotherapy (*n*, %)			0.02	0.890
Yes	127 (55.46)	63 (56.25)		
No	102 (44.54)	49 (43.75)		

### Analysis of body composition of patients with breast cancer with and without endocrine treatment

We compared the body composition of the two groups of patients with breast cancer with and without endocrine treatment. Patients who underwent endocrine treatment showed high extracellular fluid (*P* = 0.003), high water ratio (*P* = 0.004), and high basal metabolism (*P* = 0.015), and statistically significant differences were found between the above indexes of the two groups of patients. However, the differences between the two groups of patients in the rest of the indexes did not have statistical significance. (Table 6).

**Table 6:** Comparison of body composition in endocrine-treated and non-endocrine-treated groups ($\overline{{\text{x}}}\pm \mathrm{s }$)

Body composition	Endocrine therapy		*t*	*P*
	No (*n* = 112)	Yes (*n* = 229)		
Lipid (kg)	19.89 ± 5.46	19.78 ± 5.62	0.16	0.871
Bone (kg)	2.81 ± 0.23	2.85 ± 0.25	− 1.39	0.165
Protein (kg)	8.56 ± 0.89	8.70 ± 0.97	− 1.40	0.162
Total water (kg)	30.32 ± 3.16	30.86 ± 3.45	− 1.37	0.171
Intracellular fluid (kg)	19.09 ± 3.19	18.30 ± 4.49	1.87	0.062
Extracellular fluid (kg)	13.05 ± 9.23	16.65 ± 12.25	− 3.02	0.003
Water content ratio	0.42 ± 0.28	0.54 ± 0.24	− 2.87	0.004
Muscle (kg)	27.80 ± 2.96	28.58 ± 2.15	− 0.91	0.362
Basic metabolism (BMD) (J)	1186.67 ± 105.49	1216.30 ± 105.35	− 2.44	0.015
Weight (kg)	60.75 ± 8.02	61.34 ± 8.38	− 0.625	0.533
Body fat percentage (%)	31.83 ± 5.04	31.31 ± 5.58	0.86	0.393
BMI (kg/m^2^)	24.34 ± 3.23	25.23 ± 3.21	0.29	0.771
Waist–hip ratio	0.91 ± 0.06	0.91 ± 0.60	− 0.25	0.805

### Analysis of bone density-related indexes of patients with breast cancer with and without endocrine treatment.

#### Comparison of bone density-related indexes between the two groups of patients

Compared with patients without endocrine treatment, the BMD value (*P* = 0.0117), *T* value (*P* = 0.011), and BMD-related indexes were high in patients with endocrine treatment, and these indexes were statistically different between the two groups; however, the difference between the *Z* value of the two groups was not statistically significant. (Table 7).

**Table 7:** Comparison of BMD-related indices between endocrine-treated and non-endocrine-treated groups ($\overline{x}\pm \mathrm{s }$)

	Endocrine therapy		*t*	*P*
	No (*n* = 112)	Yes (*n* = 229)		
BMD (g/m^2^)	0.86 ± 0.13	0.90 ± 0.14	− 2.45	0.0117
*Z* value	0.12 ± 1.20	0.33 ± 1.29	− 1.44	0.1420
*T *value	− 0.97 ± 1.26	− 0.59 ± 1.35	− 2.58	0.0110

#### Adjustment analysis of bone density-related indexes of the two groups

To eliminate the effect of age, radiotherapy, drug type, and length of menopause on the bone density-related indexes of endocrine-treated patients with breast cancer, these factors were adjusted using multiple regression analysis, compared, and analyzed.

After adjustment, compared with patients with endocrine treatment, patients without endocrine treatment had significantly low BMD (*β* =  − 0.06, 95% CI: − 0.11 to − 0.01) (*P* < 0.01) (*P* < 0.01) and *T* value (*β* =  − 0.56, 95% CI: − 1.04 to − 0.08) (*P* < 0.05) (*P* < 0.05); however, the *Z* value was low but without statistically significant different.

Before adjustment, the BMD and *T* values of the patients in the endocrine treatment group were high. After adjusting for age, radiotherapy, drug type, and length of menopause, the BMD and *T* values of patients with endocrine treatment were low, with a statistically significant difference; however, the *Z* values of the two groups before and after adjustment showed the opposite trend and were not statistically different. (Table 8).

**Table 8:** Comparison of bone density-related indicators in adjusted endocrine-treated and non-endocrine-treated groups

	*β*	95% CI
BMD (g/m^2^)	− 0.06*	− 0.11 to − 0.01
*Z* value	− 0.48	− 0.95 to 0.01
*T* value	− 0.56*	− 1.04 to − 0.08

Note: Linear regression models were used to adjust for age, radiotherapy, type of drug, and length of menopause; patients without endocrine treatment were controls; *P < 0.05

### Analysis of body composition, bone density-related indicators, and bone metabolism indicators in patients with breast cancer undergoing different types of medication use

#### Basic conditions of participants

In this study, 229 patients with breast cancer undergoing endocrine treatment were included, of which 92 received TAM and 137 received AI, and these two groups were compared to investigate the status of body composition, bone density-related indexes, and bone metabolism indexes of patients with breast cancer who received TAM or AI.

The mean ages of the patients in the two groups were 46.6 and 56.8, and the difference was statistically significant (*P* = 0.001). The surgical modalities were mainly radical or modified radical, with the proportions of 73.9% and 71.5%, respectively. The pathologic subtype was mainly common invasive breast cancer, with proportions of 90.0% and 87.6%, respectively. The proportions of patients who received radiotherapy were 55.5% and 52.6%, respectively. The mean endocrine therapy bone density-related indexes and bone metabolism indexes of the patients who received TAM and AI were compared. The mean duration of endocrine therapy was 632.8 days and 641.4 days, respectively. Only age difference was statistically significant in the aforementioned indicators between the two groups and the rest of the indicators were not statistically significant. (Table 9).

**Table 9:** Basic profile of patients in the tamoxifen and aromatase inhibitor groups

	Tamoxifen (*n* = 92)	Aromatase inhibitor (*n* = 137)	*t*^a^/*χ*^2^	*P*
Age (years) ($\overline{x}\pm \mathrm{s }$)	46.64 ± 7.23	56.78 ± 10.18	− 8.26	0.001
Surgical procedures (*n*, %)			5.11	0.078
Radical mastoidectomy or modified radical mastoidectomy	68 (73.91)	98 (71.53)		
Breast-conserving surgery or breast reconstruction	18 (19.57)	18 (13.14)		
Simple mastectomy	6 (6.52)	21 (15.33)		
Pathological type (*n*, %)			2.31	0.316
Noninvasive breast cancer	9 (9.78)	8 (5.84)		
Nonspecific invasive breast cancer	80 (86.96)	120 (87.59)		
Specific invasive breast cancer	3 (3.26)	9 (6.57)		
Radiotherapy (*n*, %)			1.16	0.281
Yes	55 (59.78)	72 (52.55)		
No	37 (40.22)	65 (47.45)		
Duration of endocrine therapy (days) ($\overline{{\text{x}}}\pm \mathrm{s }$)	632.80 ± 444.34	641.38 ± 419.16	− 0.15	0.882

### Analysis of body composition in patients with breast cancer receiving TAM and AI

Compared with patients with breast cancer receiving TAM, patients receiving AI exhibited lower bone mass (*P* < 0.001), low protein (*P* = 0.043), low total water (*P* = 0.047), low intracellular fluids (*P* < 0.001), and low basal metabolic rate (*P* < 0.001); high extracellular fluids (*P* = 0.004), high water ratio (*P* = 0.001) and high percentage of body fat (*P* = 0.015) were high and statistically significant differences were observed for all of the above indicators. No statistically significant differences were observed for the remaining indicators. (Table 10).

**Table 10:** Comparison of body composition in tamoxifen and aromatase inhibitor groups ($\overline{x}\pm \mathrm{s }$)

Body composition	Endocrine therapy drugs		*t*	*P*
	Tamoxifen (*n* = 92)	Aromatase inhibitor (*n* = 137)		
Lipid(kg)	18.97 ± 4.92	20.33 ± 5.99	1.87	0.063
Bone (kg)	2.89 ± 0.26	2.82 ± 0.24	2.01	0.046
Protein (kg)	8.86 ± 1.00	8.60 ± 0.94	2.03	0.043
Total water(kg)	31.41 ± 3.55	30.48 ± 3.35	2.00	0.047
Intracellular fluid (kg)	19.64 ± 3.57	17.40 ± 4.82	3.80	< 0.001
Extracellular fluid (kg)	13.98 ± 10.32	18.44 ± 13.12	− 2.88	0.004
Water content ratio	0.44 ± 0.28	0.91 ± 0.26	− 3.29	0.001
Muscle (kg)	27.82 ± 12.21	29.08 ± 12.13	− 0.77	0.442
Basic metabolism (BMD) (J)	1263.51 ± 88.21	1184.60 ± 104.31	5.96	< 0.001
Weight (kg)	62.01 ± 7.78	60.88 ± 8.76	− 1.03	0.306
Body fat percentage (%)	30.23 ± 5.50	32.05 ± 5.54	− 2.45	0.015
BMI (kg/m^2^)	23.98 ± 2.68	24.40 ± 3.52	− 1.02	0.309
Waist–hip ratio	0.91 ± 0.05	0.91 ± 0.07	− 0.99	0.323

### Status of bone density-related indicators in patients with breast cancer receiving TAM and AI

#### Analysis of bone density-related indexes in the two groups of patients

Compared with the patients receiving TAM treatment, the BMD value and *T* value of the patients receiving AI are low (*P* < 0.001) and statistically different in both groups; their *Z* value was high, but the difference is not statistically significant. The results are shown in Table 11.

**Table 11:** Analysis of BMD-related indices in tamoxifen and aromatase inhibitor groups

Variables	Tamoxifen (*n* = 92)	Aromatase inhibitor (*n* = 137)	*t*	*P*
BMD	0.94 ± 0.14	0.87 ± 0.14	3.93	< 0.001
*Z* value	0.32 ± 1.25	0.34 ± 1.33	− 0.11	0.910
*T* value	− 0.19 ± 1.29	− 0.86 ± 1.33	3.76	< 0.001

#### Adjustment analysis of bone density-related indexes in two groups of patients.

To eliminate the effect of age, radiotherapy, and length of menopause on bone density-related indexes of patients with breast cancer, the aforementioned factors were adjusted by performing multiple regression analysis and then compared and analyzed.

After adjustment, compared with patients receiving TAM, patients receiving aromatase inhibitors (AI) had lower BMD (*β* = 0.08, 95% CI: 0.04–0.111) (*P* < 0.001) and *T* values (*β* = 0.69, 95% CI: 0.34–1.04) (*P* < 0.001), and the differences in BMD and *T* values were statistically significant; however, *Z* values were high and the difference was not statistically significant between the two groups.

The BMD and *T* values, which were different between the two groups before adjustment, were low and statistically significant in patients receiving AI treatment after adjustment, whereas *Z* values were high and not statistically different. (Table 12).

**Table 12:** Comparison of BMD-related indices in the adjusted tamoxifen and aromatase inhibitor groups

	*β*	95% CI
BMD (g/m^2^)	0.08***	0.04–0.11
*Z* value	− 0.02	− 0.35–0.34
*T* value	0.69***	0.34–1.04

Note: Linear regression models were used to adjust for age, duration of menopause, and duration of endocrine therapy; aromatase inhibitor (AI)-treated patients were controls; ***P < 0.001

### Status of bone metabolism indexes in patients with breast cancer receiving TAM and AI

Because not all patients with breast cancer who received endocrine therapy underwent bone metabolism index examination, we only compared and analyzed 80 patients who had previously undergone bone metabolism examination. This subset included 25 patients who received TAM endocrine therapy with 55 patients who received AI endocrine.

Compared with patients receiving TAM, the bone metabolism indexes of patients receiving aromatase inhibitor (AI) class of drugs were high in osteocalcin (*P* < 0.001), total type I collagen amino acid extended peptide (*P* < 0.001), total 25-hydroxyvitamin D (*P* = 0.041), and *β*-collagen specific sequence (*P* = 0.001). The residual indexes of bone alkaline phosphatase were high and parathyroid hormone (entire segment) was low, but the difference between the two groups was not statistically significant. The results are shown in Table 13.

**Table 13:** Comparison of bone metabolism indexes between the two groups of patients

Bone metabolic index	TAM (*n* = 25)	AI (*n* = 55)	*t*	*P*
Bone alkaline phosphatase (μg/mL)	20.18 ± 17.68	23.05 ± 10.84	− 0.89	0.375
Osteocalcin (μg/mL)	11.06 ± 2.44	17.30 ± 7.47	− 4.07	< 0.001
Total *N*-terminal propeptide of type I collagen (ng/mL)	33.75 ± 13.44	53.56 ± 30.09	− 4.07	< 0.001
Total 25-hydroxy-vitamin-D (ng/mL)	14.18 ± 4.09	16.73 ± 6.76	− 2.08	0.041
Intact parathyroid hormone (ng/mL)	55.66 ± 30.24	55.17 ± 2 5.49	0.08	0.944
Β-Crosslaps (μg/mL)	0.29 ± 0.14	0.44 ± 0.25	− 3.43	0.001

### Evaluation of the machine learning model

**Fig. 1:**
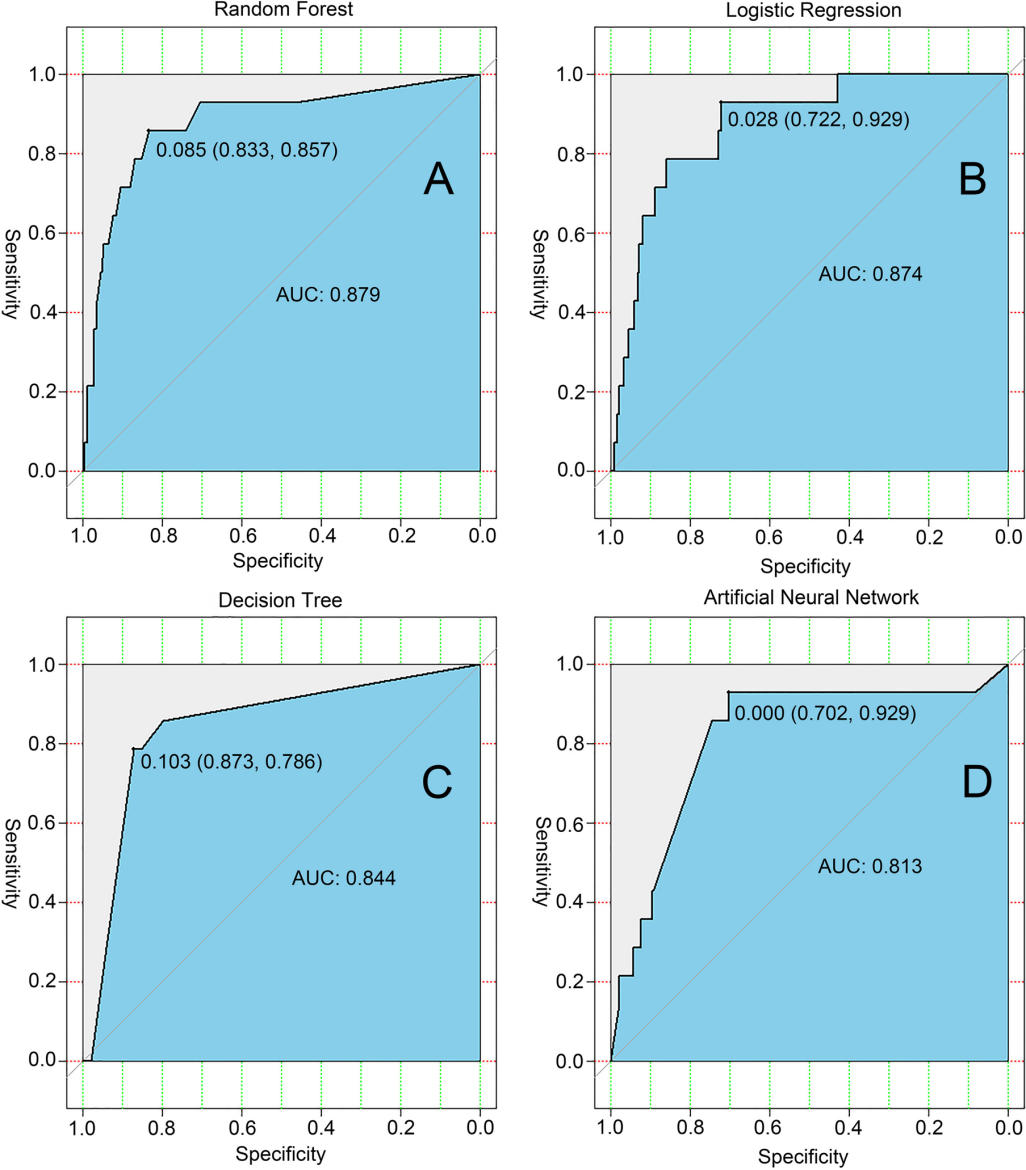
Machine learning models predicting osteoporosis occurrence in patients with breast cancer and healthy women in the test set; **A** ROC curve for the random forest model; **B** ROC curve for the logistic regression model; **C** ROC curve for the decision tree model; **D** ROC curve for the artificial neural network model; *ROC* receiver operating characteristic curve; *AUC* area under the curve

**Fig. 2:**
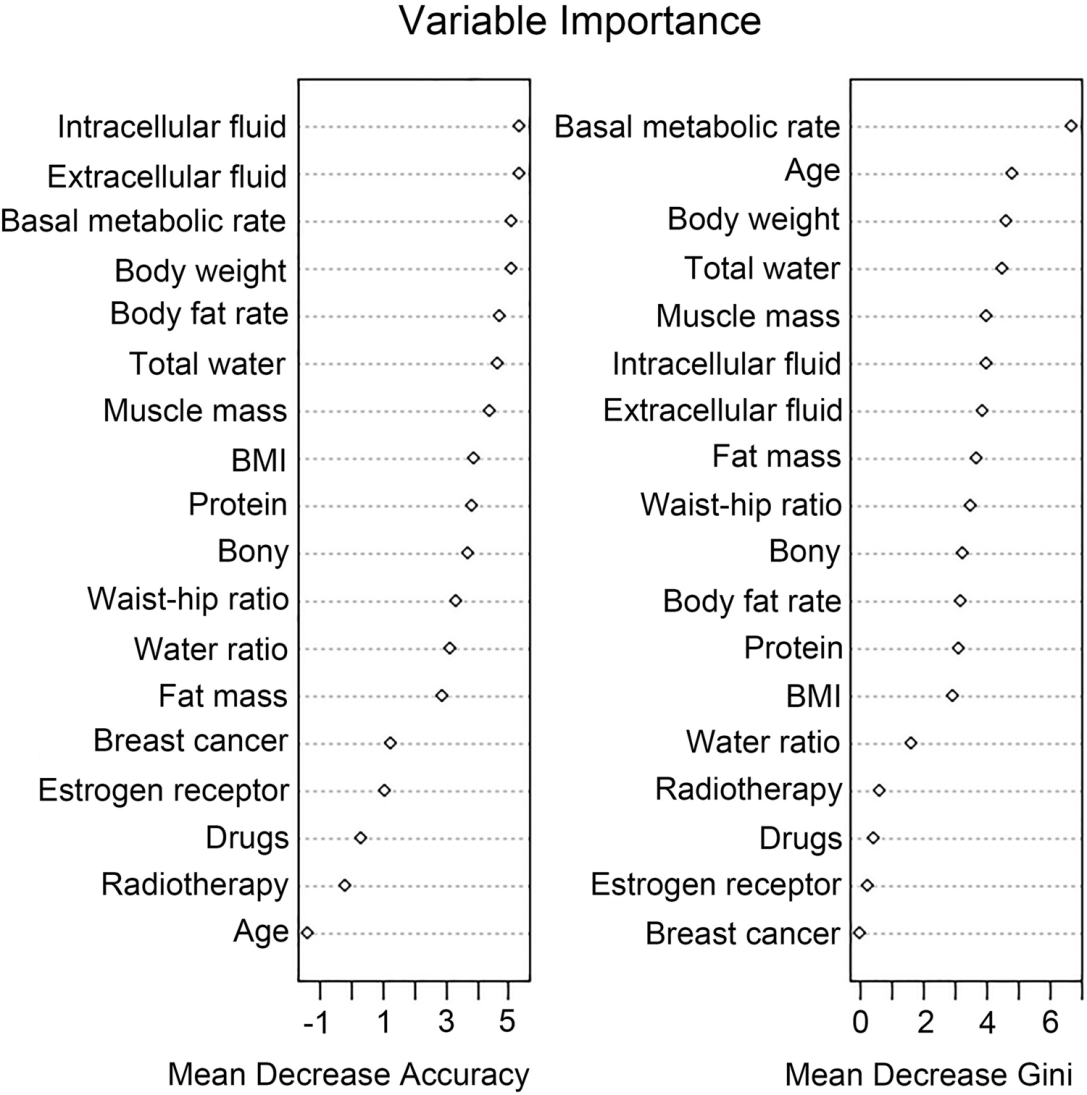
Ranking of the importance of the variables included in the random forest model

Our results showed that there is an association between body composition and breast cancer, and the treatment of breast cancer with bone loss, which has not been reported in previous studies. We randomly divided the study subjects into the training and test sets in a ratio of 7:3 and used four machine learning algorithms to create predictive models to assess the risk of incidence of osteoporosis in patients with breast cancer and healthy women. For the test set, we plotted the predicted receiver operating characteristic curves and calculated their AUC for model evaluation and comparison. The RF algorithm model showed the highest accuracy and best model performance in predicting the occurrence of osteoporosis in patients with breast cancer and healthy women (test set: AUC = 0.879) (Fig. 1A), compared with that of the LR (test set: AUC = 0.874) (Fig. 1B), ID3 (test set: AUC = 0.844) (Fig. 1C), and ANN models (test set: AUC = 0.813) (Fig. 1D). Additionally, we ranked the importance of the variables included in the RF model (Fig. 2) and found that the intracellular fluid, extracellular fluid, basal metabolic rate, body weight, and body fat percentage were the five most important factors affecting the occurrence of osteoporosis in patients breast cancer and healthy women. Altogether, these results suggested a strong association between body composition and the occurrence of osteoporosis.

## Discussion

In this study, comparative analyses were performed among three sets of populations, namely the health check-up women population and non-endocrine-treated patients with breast cancer, non-endocrine-and endocrine-treated patients, and patients treated with TAM and AIs. The analyses revealed that alterations in body composition and BMD occurred in all three sets.

The main changes in the body composition of patients with breast cancer who were not treated with endocrine therapy compared with that of the healthy women were as follows: reduction in nonadipose tissue; increased risk of edema; increased percentage of overweight or obesity, and altered fat distribution, as indicated by low bone mass, protein, body fat percentage, muscle, and basal metabolism; and high total water, intracellular fluids, extracellular fluids, water ratios, BMI, and waist-to-hip ratios. Compared with non-endocrine-treated patients, the patients in the endocrine-treated group had a higher risk of edema, as indicated by high extracellular fluid, water ratio, and basal metabolism. Compared to the patients with breast cancer receiving TAM, the body compositions of patients receiving AI analogs showed predominant changes of increased fat distribution and an increased risk of edema development, as indicated by low intracellular fluid content, including low bone, protein, total water, intracellular fluid, and basal metabolic ratios, and a high extracellular fluid content, including water ratio and body fat percentage.

This study showed that an endocrine therapy-induced decrease in estrogen levels may cause changes in body composition. This observation is consistent with the results of a genetic-level study by Napoli et al. (Napoli et al. 2013), where the effect of the rs700518 polymorphism in the CYP19A1 gene was identified on changes in body composition in postmenopausal patients with ER-positive breast cancer undergoing AI therapy, and with those of a clinical-level study by Liu et al. (Liu et al. 2022), who examined the association of the body composition regarding clinical prognosis in Chinese female patients with breast cancer; visceral obesity was associated with higher disease recurrence rates, and sarcopenia was significantly associated with increased recurrence rates and increased overall mortality. It is believed that physical activity and dietary changes during chemotherapy cause a decrease in nonadipose tissue in patients, which can be related to the fact that the patients enrolled in this study had already undergone stages of surgery, radiotherapy, and chemotherapy. In postmenopausal patients with breast cancer, the adipose tissue is the main site of estrogen production and aromatase expression, suggesting that body fat content may be related to the efficacy of AI (Baglia et al. 2019). In an animal study of endocrine therapy for breast cancer, endocrine therapy was found to be associated with fat accumulation and increased preadipocytes, and in its translational study, it was found that TAM was associated with large-diameter breast adipocytes in women with obesity and that endocrine therapies may disrupt adipocytoblasts and support adipocyte hypertrophy, which may lead to ectopic lipid deposition (Scalzo et al. 2021). The water ratio of a healthy human body is generally 0.36–0.39, and the high water ratio in the body composition of patients with breast cancer in this study may be related to the poor return of lymphatic fluid to the upper extremities after the dissection of the axillary lymph node in patients with breast cancer, in addition to the factor of receiving endocrine therapy.

In addition to the changes in body composition, bone density also changes in patients with breast cancer. Compared with the healthy women, non-endocrine-treated patients had low *Z*- and *T* values for BMD, indicating high proportions of both bone loss (45.5% of patients) and osteoporosis (10.7% of patients). Endocrine-treated patients with breast cancer had low BMD and *Z*-scores, which suggested high proportions of osteopenia and osteoporosis, and an increase in bone alkaline phosphatase (BAP), a marker of bone formation. Compared with TAM-treated patients, AI-treated patients had low BMD and *T* values, indicating high proportions of bone loss and osteoporosis, and low levels of bone formation markers such as OC, procollagen type I N-propeptide, and total 25-hydroxyvitamin D in bone metabolism indexes in AI-treated patients, along with low levels of the bone resorption indicator *β*-collagen specific sequence (*β*-CTX).

The reason for this change may be that lower estrogen levels suppress osteoblast activity and increase osteoclast activity, accelerating bone resorption, which is consistent with the findings of Locquet et al. (2017), who reported that 69% of patients with breast cancer in their study had lumbar spine bone loss and osteoporosis, indicating that bone loss is more pronounced in patients with breast cancer after comprehensive treatment (such as chemotherapy and endocrine therapy) and that the treatment received may lead to the development of the clinical signs of reduced BMD, bone loss, and osteoporosis in patients. Several clinical studies have confirmed that the use of TAM and AI has increased the risk of osteoporosis and fractures, resulting in an increase in patient disability and mortality, such as inducing an earlier appearance (Edwards et al. 2011), increased incidence of hip fracture (Goldvaser et al. 2018), a notable decrease in quality of life, and reduced treatment adherence (Pineda-Moncusí et al. 2019), all of which seriously affects the overall prognosis of patients with breast cancer. Moreover, the associated treatment can cause a heavy financial burden and psychological pressure on patients, as well as a considerable economic burden on society. Kuba et al. (2021) used high-resolution peripheral quantitative computed tomography to assess BMD and bone microstructural changes in patients with early-stage breast cancer undergoing AI treatment. In the Arimidex, Tamoxifen, Alone or in Combination (ATAC) study of anastrozole, after 5 years of administration, the lumbar spine and total hip BMD decreased by 6.1% and 7.2%, respectively, with the loss occurring rapidly in the first 2 years of administration and being more pronounced in early menopausal patients (Cuzick et al. 2010).

Bone metabolism indexes reflect the level of osteoblast activity and bone matrix metabolism. The commonly used bone metabolism indices in clinical practice include BAP, OC, total collagen type I amino acid extended peptide (RINP), total 25-hydroxyvitamin D (1,25[OH]2D3), parathyroid hormone (PTH), and *β*-CTX. In this study, the possible reason for the altered bone metabolism indexes is that estrogen affects bone metabolism through pathways such as decreasing bone sensitivity to PTH and decreasing calcitonin production, whereas increased bone resorption disrupts the equilibrium between bone formation and bone resorption, mentioning that there is a reactive increase in bone formation. The International Osteoporosis Foundation recommends the use of RINP and *β*-CTX in the prediction of fracture risk and monitoring of osteoporosis treatment (Cuzick et al. 2010). Similarly, Japanese scholars pointed out in the Guidelines for the Use of Bone Transformation Indicators in the Diagnosis and Treatment of Osteoporosis by the Japanese Osteoporosis Society (2012 edition) that, in practice, bone metabolism indexes help identify primary and secondary osteoporosis, predicting the rate of bone loss, evaluating the risk of fracture, understanding the progression of the disease, selecting interventions, and monitoring the efficacy of medications and adherence of patients to those medications, and that they are a good means of dynamic clinical surveillance and detection (Bauer et al. 2012; Nishizawa et al. 2013).

Therefore, we constructed a machine learning model to predict the risk of osteoporosis in patients with breast cancer and healthy women using parameters such as body composition, age, hormone receptor status, and medication type. The evaluation of our model showed high accuracy in predicting osteoporosis, reflecting the predictive value of the model. The prediction accuracy was significantly higher than common algorithms such as ID3, LR, and ANNs. By ranking the importance of the model parameters, it was found that intracellular fluid, extracellular fluid, basal metabolism, body weight, and body fat percentage were the five most important factors affecting bone mass in the study subjects. The findings of this study suggest that in clinical settings, the changes in body composition of patients with breast cancer can be observed to determine the bone density status and provide a scientific and accurate method to prevent the occurrence of osteoporosis.

The following study limitations should be noted when interpreting the results of this study: (1) As the study site was limited to one healthcare organization, the sample selection lacked some representativeness, which may affect the generalizability of the findings. Therefore, caution should be exercised when replicating it in other populations. (2) Due to the limited sample size, the stratification of different endocrine therapy medications was not detailed enough. (3) This study only examined some of the laboratory indicators, and the monitoring of the indicators was not continuous, failing to describe the dynamic trends of body composition, bone density and bone metabolism indicators during endocrine therapy in breast cancer patients. (4) Due to the limitations of the cross-sectional study design, it is not possible to make effective causal inferences, but the results of the current study suggest that there may be an association between endocrine therapy and body composition and bone mineral density in breast cancer patients. (5) Although the prediction model developed in this study is based on body composition measures and some clinical characteristics of breast cancer patients, the data are easily accessible and non-invasive, and the model has a satisfactory predictive efficiency, it should be cautiously replicated in other populations due to the limitations of the above study.

## Supplementary Information

Below is the link to the electronic supplementary material.

Supplementary file1 (XLSX 64 KB)

Supplementary file2 (XLSX 52 KB)

Supplementary file3 (DOCX 13 KB)

## Data Availability

All data can be obtained in the supplementary files.
