# Supplementary material for: Machine learning predicts the risk of osteoporosis in patients with breast cancer and healthy women
Source: J Cancer Res Clin Oncol. 2024 Feb 23;150(2):102. doi: 10.1007/s00432-024-05622-8 (PMC10891247; doi:10.1007/s00432-024-05622-8)
Supplement: Supplementary file 3 — Supplementary file3 (DOCX 13 KB) [file 432_2024_5622_MOESM3_ESM.docx]

rm(list=ls())

setwd(dir="c:/Users/a/Desktop/")

library(pROC)

seer<-read.csv("a.csv",header = TRUE)

set.seed(70)

seer<-seer[complete.cases(seer),]

seer<-na.omit(seer)

train_sub = sample(nrow(seer),7/10*nrow(seer))

train_data = seer[train_sub,]

test_data = seer[-train_sub,]

train_data$group = as.factor(train_data$group)

test_data$group = as.factor(test_data$group)

#ANN

library("nnet")

fit_nnet=nnet(group~.,data=train_data,size=4,decay=5e-4,maxit=200)

nnet_test <- predict(fit_nnet,test_data,type="raw")

roc.glm <- roc(test_data$group, as.numeric(nnet_test))

plot(roc.glm, print.auc=TRUE, auc.polygon=TRUE, grid=c(0.1, 0.2),grid.col=c("green", "red"), max.auc.polygon=TRUE,auc.polygon.col="skyblue", print.thres=TRUE,main='ANN ROC')

#RF

library("randomForest")

randomforest <- randomForest(group ~ .,

data = train_data,

ntree =300,

mtry=4,

importance=TRUE ,

proximity=TRUE)

varImpPlot(randomforest, main = "variable importance")

pre_ran <- predict(randomforest,newdata=test_data,type = 'response')

ran_roc <- roc(test_data$group,predict(randomforest,newdata=test_data,type="prob")[,2])

plot(ran_roc, print.auc=TRUE, auc.polygon=TRUE, grid=c(0.1, 0.2),grid.col=c("green", "red"), max.auc.polygon=TRUE,auc.polygon.col="skyblue",print.thres=TRUE,main='RFROC,mtry=3,ntree=500')

print(randomforest)

#LR

library(caTools)

glm.train <- glm(as.factor(train_data$group) ~ ., data = train_data,family = binomial)

glm.test <- predict(glm.train, newdata = test_data, type = "response")

roc.glm <- roc(test_data$group5, as.numeric(glm.test))

plot(roc.glm, print.auc=TRUE, auc.polygon=TRUE, grid=c(0.1, 0.2),grid.col=c("green", "red"), max.auc.polygon=TRUE,auc.polygon.col="skyblue", print.thres=TRUE,main='LR ROC')

#predict=ifelse(glm.test>0.5,1,0)

true_label=test_data$group5

error=predict-true_label

accuracy=(nrow(test_data)-sum(abs(error)))/nrow(test_data)

#ID3

library(rpart)

library("rpart.plot")

seer_decisiontree_ID3 <- rpart(group ~ .,

data = train_data,

method="class",

parms=list(split="information"))

printcp(seer_decisiontree_ID3)

seer_decisiontree_ID3_prune<-prune(seer_decisiontree_ID3,cp= 0.01)

decisiontree_roc_ID3 <- roc(test_data$group,predict(seer_decisiontree_ID3_prune,newdata=test_data,type="prob")[,2])

plot(decisiontree_roc_ID3,print.auc=TRUE, auc.polygon=TRUE, grid=c(0.1, 0.2),grid.col=c("green", "red"), max.auc.polygon=TRUE,auc.polygon.col="skyblue", print.thres=TRUE,main='ID3ROC')
